# Thrombospondin 2 as a Predictive Biomarker for HCC in Hepatitis C Patients: A Longitudinal Study Following DAA Therapy

**DOI:** 10.1111/jvh.14025

**Published:** 2024-10-15

**Authors:** Takanobu Iwadare, Takefumi Kimura, Ayumi Sugiura, Taiki Okumura, Shun‐ichi Wakabayashi, Hiroyuki Kobayashi, Yuki Yamashita, Tomoo Yamazaki, Satoru Joshita, Naoki Tanaka, Takeji Umemura

**Affiliations:** ^1^ Division of Gastroenterology and Hepatology, Department of Medicine Shinshu University School of Medicine Matsumoto Nagano Japan; ^2^ Consultation Center for Liver Diseases Shinshu University Hospital Matsumoto Nagano Japan; ^3^ Department of Internal Medicine Sato Hospital Nakano Nagano Japan; ^4^ Department of Medicine University of California San Diego La Jolla California USA; ^5^ Department of Internal Medicine Yodakubo Hospital Nagawa Nagano Japan; ^6^ Department of Global Medical Research Promotion Shinshu University Graduate School of Medicine Matsumoto Nagano Japan; ^7^ International Relations Office Shinshu University School of Medicine Matsumoto Nagano Japan; ^8^ Research Center for Social Systems Shinshu University Matsumoto Nagano Japan

**Keywords:** biomarker, direct‐acting antivirals, hepatitis C virus, hepatocellular carcinoma, thrombospondin 2

## Abstract

This multicentre study investigated the dynamics of thrombospondin 2 (TSP2) levels during direct‐acting antiviral (DAA) therapy in hepatitis C virus (HCV) infected patients and evaluated TSP2's potential as a predictive marker for hepatocellular carcinoma (HCC). All 134 participants achieved sustained virological response at 12 weeks (SVR12) with DAA therapy, and serum TSP2 levels significantly decreased from before and after treatment (*p* < 0.001). During the median follow‐up period of 6.0 years, HCC after DAA therapy was observed in 16 patients (11.9%). Patients with serum TSP2 High (≥ 32 ng/mL) at SVR12 had a significantly higher cumulative occurrence of HCC than did those without (26.5% vs. 7.0%, *p* = 0.0033). A multivariate Cox proportional hazards model identified male gender (HR 4.84, *p* = 0.005), HCC history (HR 4.61, *p* = 0.017) and TSP2 High (HR 3.93, *p* = 0.009) as significant independent predictors of HCC occurrence after DAA therapy. The model had a high concordance index of 0.878. Additionally, combining TSP2 High and FIB‐4 High (≥ 3.538) at SVR12 yielded high specificity and negative predictive value (0.941 and 0.917, respectively) for predicting HCC. Kaplan–Meier analysis showed a higher HCC incidence in the TSP2 High + FIB‐4 High group (log‐rank *p* < 0.0001). In conclusion, TSP2 may be a promising biomarker for personalised HCC surveillance in DAA‐treated hepatitis C patients.

**Abbreviations**
AFP: alpha‐fetoprotein
ALP: alkaline phosphatase
ALT: alanine aminotransferase
APRI: aspartate aminotransferase to platelet ratio index
AST: aspartate aminotransferase
AUROC: area under the receiver operating characteristic curve
CI: confidence interval
*C*‐index: concordance index
DAA: direct‐acting antiviral
DM: diabetes mellitus
EOT: end of treatment
FIB‐4: fibrosis‐4 index
HCC: hepatocellular carcinoma
HCV: hepatitis C virus
HR: hazard ratio
HSC: hepatic stellate cell
IFN: interferon
IQR: interquartile range
Plt: platelet count
PPV: positive predictive value
SVR: sustained virological response
SVR12: sustained virological response at 12 weeks
TSP2: thrombospondin 2
WBC: white blood cell count

## 1 Introduction

In 2020, approximately 56.8 million viraemic hepatitis C virus (HCV) infections were estimated worldwide [1]. HCV infection is a significant risk factor for the occurrence of hepatocellular carcinoma (HCC) [2, 3]. Many patients with HCV have experienced significant benefits from revolutionary direct‐acting antiviral (DAA) therapies towards viral elimination [4]. However, the risk of HCC persists even after viral clearance, underscoring the need to identify individuals at both high and low risk of HCC development and establish suitable follow‐up intervals [5].

Encoded by the THBS2 gene, the thrombospondin 2 (TSP2) protein plays a crucial role in various biological processes, including collagen/fibrin formation, bone growth, maintenance of normal vessel density, haemostasis and cell adhesion [6]. We earlier showed a correlation between serum TSP2 levels and the severity of liver tissue fibrosis and inflammation in patients with HCV [7]. Matsumae et al. reported on the usefulness of serum TSP2 for predicting HCC development after a sustained virological response (SVR) in a HCC‐naïve cohort [8]. However, the precise impact of DAA treatment on TSP2 levels remains unknown, and there is a lack of understanding on the predictive performance of TSP2 for carcinogenesis in real‐world HCV cohorts that include patients with a history of HCC. To clarify these issues, we herein show the continuous reduction of TSP2 levels by DAA treatment and demonstrate the utility of TSP2 as a predictive marker for HCC development after DAA therapy among hepatitis C patients with and without prior HCC.

## 2 Methods

### 2.1 Patients and Clinical Examinations

In this multicentre, prospectively registered, retrospective cohort analysis study conducted across Nagano prefecture, Japan, patients with HCV underwent DAA therapy at Shinshu University Hospital (Matsumoto, Japan) or its affiliated institutions between October 2014 and September 2017. A total of 960 patients with chronic HCV infection were registered upon commencing DAAs for age, gender, prior interferon (IFN) treatment and history of an HCC complication. After excluding cases lacking sufficient clinical data or serum samples for analysis, 134 HCV patients were ultimately enrolled in this study. Written consent forms were obtained from all patients. The ethnic background of all patients was Asian. The diagnosis of HCV infection was based on previously reported criteria as the presence of serum HCV antibodies and detectable HCV RNA [9]. All patients were negative for hepatitis B surface antigen as well as antibodies to hepatitis B core antigen and the human immunodeficiency virus. No patients complicated with active HCC were included. Patients who were diagnosed as having alcoholic liver disease, defined as an average daily ethanol consumption of > 60 g for men and > 40 g for women, were excluded. Patients with evidence of other liver disease, such as primary biliary cholangitis or autoimmune hepatitis, were excluded as well. This study was reviewed and approved by the Institutional Review Board of Shinshu University Hospital (approval number: 3021) and conducted according to the principles of the Declaration of Helsinki.

The study patients were treated with DAA regimens that included daclatasvir + sunaprevir for 24 weeks, ledipasvir/sofosbuvir, ombitasvir/paritaprevir/ritonavir, or elbasvir + grazoprevir for 12 weeks for HCV genotype 1, or sofosbuvir + ribavirin for 12 weeks for genotype 2 based on guidelines from the Japan Society of Hepatology [10]. Sustained virological response at 12 weeks (SVR12) was defined as undetectable HCV RNA at 12 weeks after the completion of DAA therapy.

Diabetes mellitus (DM) was defined as meeting any of the following criteria: an HbA1c level of 6.5% or higher, a fasting blood glucose level of 126 mg/dL or higher, or the current use of antidiabetic medication [11, 12]. Fibrosis‐4 index (FIB‐4) and aspartate aminotransferase (AST)‐to‐platelet ratio index (APRI) were calculated according to the following formulae: FIB‐4 = (age [years] × AST [IU/L])/(platelet count [Plt] [10^9^/L] × alanine aminotransferase [ALT] [IU/L]^1/2^) [13] and APRI = (AST/upper limit of normal; 28 [U/L]) × (100/Plt [10^9^/L]) [14]. Serum TSP2 concentrations were determined using enzyme‐linked immunosorbent assays (Quantikine ELISA, #DTSP20, R&D Systems, Minneapolis, MN) [7, 15, 16]. All serum samples were immediately stored at −30°C until testing.

### 2.2 Patient Follow‐Up

Patients were regularly monitored at intervals of at least 6 months using ultrasonography or computed tomography, at which time serum alpha‐fetoprotein (AFP) levels were measured as well. Cirrhotic patients underwent more frequent evaluations, with assessments scheduled at least every 3–4 months [17, 18]. The radiological diagnosis of HCC was based on the American Association for the Study of Liver Diseases practice guidelines on the management of HCC as either: (1) the presence of a hepatic lesion > 2 cm in diameter with typical vascular pattern for HCC on one dynamic imaging technique or AFP > 200 ng/mL, or (2) the presence of a lesion 1–2 cm in diameter with typical vascular pattern for HCC on two dynamic imaging techniques.

Follow‐up time was defined as the number of years from SVR12 to HCC diagnosis or from SVR12 to the last follow‐up visit when protocol surveillance confirmed no event.

### 2.3 Statistical Analysis

Clinical data were expressed as the number (percentage) or the median (interquartile range [IQR]). Statistical analyses were performed using R software ver. 4.3.0. The Mann–Whitney *U* test and Chi‐squared test were employed for comparisons between the study groups. Wilcoxon matched‐pairs signed‐rank testing was used for evaluating parameters before DAA treatment and at SVR12. Diagnostic accuracy was evaluated using the area under the receiver operating characteristic curve (AUROC). The Youden index identified cut‐off values, with the nearest clinically applicable value to the cut‐off considered the optimal threshold for clinical convenience. Comparisons of the AUROC were performed using the DeLong test. The Kaplan–Meier method and log‐rank testing were employed to estimate disease progression. The Cox proportional hazards model was adopted to assess univariate and multivariate covariates for the occurrence of HCC. All statistical tests were two‐tailed and evaluated at the 0.05 level of significance.

## 3 Results

### 3.1 Changes in Clinical Characteristics During DAA Treatment

The clinical features of the 134 HCV‐infected patients before DAA treatment and at SVR12 are summarised in Table 1. Prior to therapy, median age was 68 years, with 58 patients (43.3%) being male. 22 patients (16.4%) had DM, 26 patients (19.4%) had cirrhosis, 46 patients (34.3%) had received IFN treatment, and 13 patients (9.7%) had a history of HCC. Median haemoglobin decreased slightly by DAA treatment (14.0–13.8 g/dL, *p* < 0.003), while white blood cell count (WBC) and Plt both increased (WBC: 4580–4830 μ/L; Plt: 15.6–16.8 × 10^4^/μL, both *p* < 0.001).

**TABLE 1:** Clinical features and laboratory data of HCV‐infected patients before DAA therapy and at SVR12.

	Before DAA therapy (median, IQR)/*n* (%)	SVR12 (median, IQR)	*p*
Age (years)	68 (60–74)	—	—
Male	58 (43.3%)	—	—
DM	22 (16.4%)		
HCV serotype (1/2)	118 (88.1%)/16 (11.9%)	—	—
Cirrhosis	26 (19.4%)	—	—
Prior IFN	46 (34.3%)	—	—
HCC history	13 (9.7%)	—	—
DAA regimen			
DCV/ASV	43 (32.1%)	—	—
LDV/SOF	54 (40.3%)	—	—
OBV/PTV/r	2 (1.5%)	—	—
EBV/GRZ	16 (11.9%)	—	—
SOF/RBV	19 (14.2%)	—	—
WBC (μ/L)	4580 (3608–5388)	4830 (3915–5970)	**< 0.001**
Hb (g/dL)	14.0 (12.8–15.3)	13.8 (12.6–15.1)	**0.003**
Plt (×10^4^/μL)	15.6 (11.2–19.4)	16.8 (12.4–20.6)	**0.001**
Alb (g/dL)	4.1 (3.9–4.3)	4.2 (4.0–4.5)	**< 0.001**
AST (U/L)	41 (29–54)	24 (19–29)	**< 0.001**
ALT (U/L)	37 (25–57)	16 (13–21)	**< 0.001**
AFP (ng/mL)	4.6 (2.6–7.9)	3.4 (2.1–4.8)	**< 0.001**
FIB‐4	2.86 (1.93–4.32)	2.37 (1.70–3.66)	**< 0.001**
APRI	0.70 (0.41–1.12)	0.48 (0.33–0.75)	**< 0.001**
TSP2 (ng/mL)	29.1 (22.3–43.7)	24.6 (18.5–32.1)	**< 0.001**

*Note:* Bold indicates a *p* value of less than 0.05. Abbreviations: AFP, alpha‐fetoprotein; Alb, albumin; ALT, alanine aminotransferase; APRI, aspartate aminotransferase to platelet ratio index; AST, aspartate aminotransferase; ASV, asunaprevir; DAA, direct‐acting antiviral; DCV, daclatasvir; DM, diabetes mellitus; EBV, elbasvir; FIB‐4, fibrosis‐4 index; GRZ, grazoprevir; Hb, haemoglobin; HCC, hepatocellular carcinoma; HCV, hepatitis C virus; IFN, interferon; IQR, interquartile range; LDV, ledipasvir; OBV, ombitasvir; Plt, platelet count; PTV, paritaprevir; RBV, ribavirin; r, ritonavir; SOF, sofosbuvir; SVR12, sustained virological response at 12 weeks; TSP2, thrombospondin 2; WBC, white blood cell count.

Albumin (Alb) level increased from a median of 4.1 to 4.2 g/dL (*p* < 0.001). The liver enzyme levels of AST and ALT decreased from 41 to 24 and 37 to 16 U/L, respectively (both *p* < 0.001). The liver fibrosis markers FIB‐4 and APRI were also reduced by treatment from median values of 2.86 to 2.37 and 0.70 to 0.48, respectively (both *p* < 0.001). These results demonstrated improvements in liver function and liver fibrosis markers, indicating favourable outcomes for DAA treatment in the management of HCV infection.

### 3.2 Changes in Serum TSP2 Levels During DAA Treatment

**FIGURE 1:**
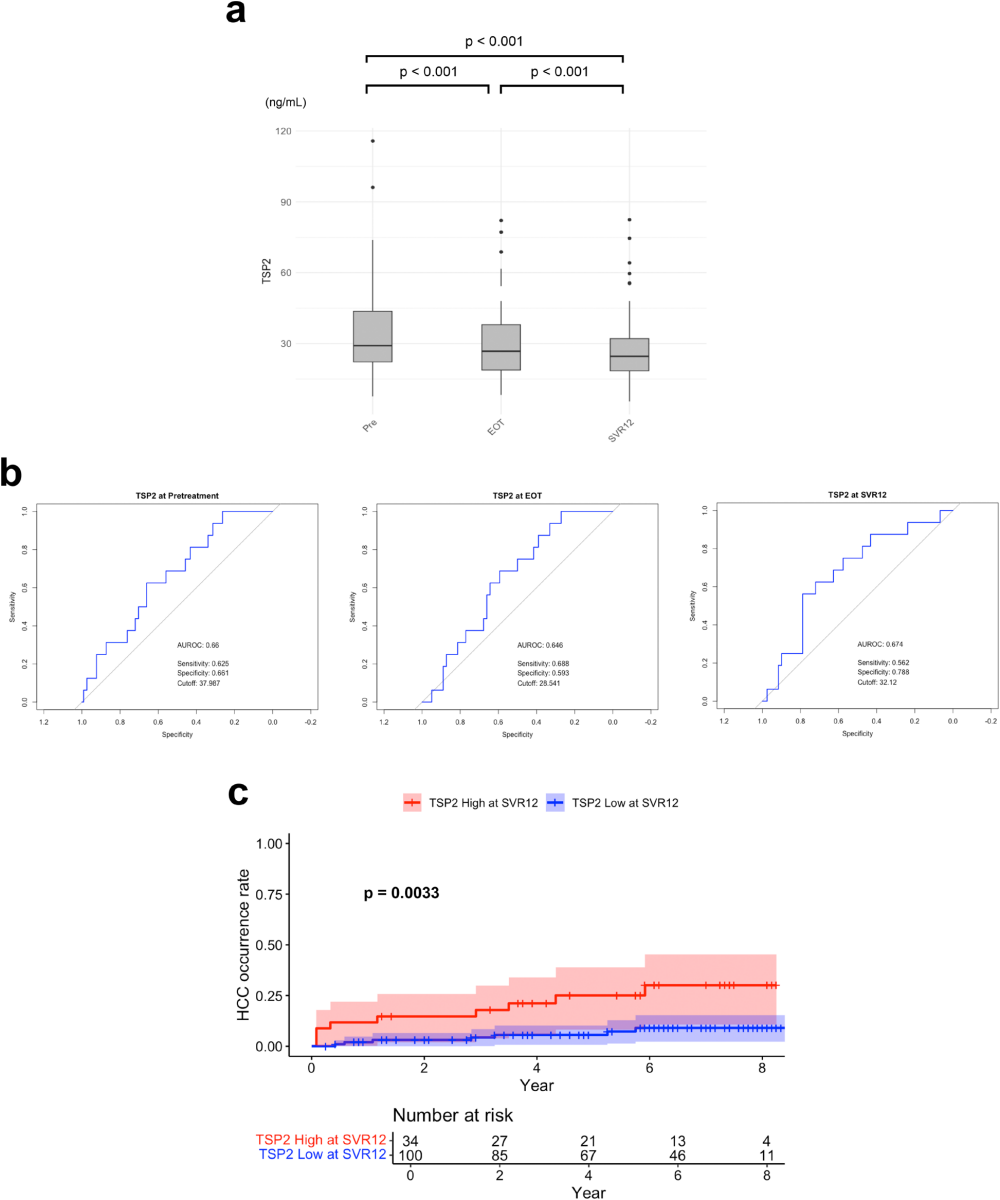
(a) Comparisons of serum TSP2 levels at pretreatment, EOT and SVR12. (b) Receiver operating characteristic analysis of TSP2 levels for HCC at pretreatment, EOT and SVR12. (c) Cumulative HCC occurrence rate after DAA therapy stratified by TSP2 levels. TSP2 High: TSP2 > 32.12 ng/mL. The ‘Year’ axis starts from SVR12. AUROC, area under the receiver operating characteristic curve; DAA, direct‐acting antiviral; EOT, end of treatment; HCC, hepatocellular carcinoma; Pre, pretreatment; SVR12, sustained virological response at 12 weeks; TSP2, thrombospondin 2.

We next investigated the alterations in serum TSP2 levels in hepatitis C patients before DAA treatment, at the end of treatment (EOT), and at SVR12 (Figure 1a).

TSP2 level exhibited a significant decrease from pretreatment to EOT (34.4–26.8 ng/mL, *p* < 0.001). A further significant decrease in serum TSP2 level was seen from EOT to SVR12 (26.8–24.6 ng/mL, *p* < 0.001).

### 3.3 Serum TSP2 Levels and Cumulative HCC Incidence After DAA Therapy

The median follow‐up evaluation period for the 134 patients was 6.0 years (IQR: 3.9–7.4 years). 16 patients (11.9%; 12 male and 4 female) reached the outcome of HCC occurrence after DAA therapy.

The AUROC for TSP2 in predicting HCC occurrence after DAA therapy was 0.660 at pretreatment, 0.646 at EOT and 0.674 at SVR12 (Figure 1b). There were no significant differences between pretreatment and EOT (*p* = 0.93), pretreatment and SVR12 (*p* = 0.483), or EOT and SVR12 (*p* = 0.468). The cut‐off value for TSP2 was 37.987 ng/mL at pretreatment, 28.541 ng/mL at EOT, and 32.12 ng/mL at SVR12, with sensitivity and specificity results at each cut‐off value being 0.625 and 0.661, 0.688 and 0.593, and 0.562 and 0.788, respectively. Although no statistically significant differences were observed, the TSP2 level at SVR12 exhibited the highest AUROC, sensitivity, and specificity for predicting HCC occurrence after DAA treatment. Consequently, it was selected for use in subsequent analyses. Kaplan–Meier survival analysis using the cut‐off value for TSP2 at SVR12 revealed a significantly higher rate of HCC occurrence post‐SVR12 in the high TSP2 group (log‐rank *p* = 0.0033) (Figure 1c). TSP2 also appeared to be predictive for carcinogenesis even shortly after DAA treatment.

### 3.4 Comparison of Clinical Characteristics Between the Non‐HCC and HCC Occurrence Groups at SVR12

To identify the predictors of HCC occurrence after DAA therapy, we compared the clinical characteristics at SVR12 between non‐HCC occurrence and HCC occurrence patients (Table 2). Patients with HCC occurrence after DAA therapy had significantly higher age (*p* = 0.032), prevalence of cirrhosis (*p* = 0.004), history of HCC (*p* < 0.001), AST level (*p* = 0.012), FIB‐4 (*p* = 0.004), APRI (*p* = 0.006) and TSP2 (*p* = 0.024), along with significantly lower Alb level (*p* = 0.023), versus non‐HCC occurrence patients.

**TABLE 2:** Comparison of clinical characteristics at SVR12 between non‐HCC occurrence patients and HCC occurrence patients.

	Non‐HCC (*n* = 118) (median, IQR)/n (%)	HCC (*n* = 16) (median, IQR)/n (%)	*p*
Age (years)	69 (63–75)	71 (68–74)	**0.032**
Male	63 (53.3%)	12 (75.0%)	0.166
DM	21 (17.8%)	1 (6.3%)	0.253
HCV serotype (1/2)	103 (87.3%)/15 (12.7%)	15 (93.8%)/1 (6.3%)	0.458
Cirrhosis	19 (16.1%)	8 (50.0%)	**0.004**
Prior IFN	50 (42.4%)	10 (62.5%)	0.096
HCC history	7 (5.9%)	8 (50.0%)	**< 0.001**
WBC (μ/L)	4880 (3975–6245)	3860 (3293–4420)	0.334
Hb (g/dL)	14.0 (12.6–15.4)	13.5 (12.9–13.9)	0.514
Plt (×10^4^/μL)	16.8 (13.6–19.8)	11.7 (10.6–13.9)	0.077
Alb (g/dL)	4.3 (4.1–4.5)	4.0 (3.8–4.2)	**0.023**
AST (U/L)	24 (19–29)	30 (28–34)	**0.012**
ALT (U/L)	16 (13–26)	19 (15–32)	0.102
FIB‐4	2.46 (1.66–3.16)	4.20 (3.95–5.00)	**0.004**
APRI	0.48 (0.33–0.75)	0.85 (0.73–1.07)	**0.006**
TSP2 (ng/mL)	24.1 (18.2–31.6)	33.1 (28.8–37.0)	**0.024**

*Note:* Bold indicates a *p* value of less than 0.05. Abbreviations: Alb, albumin; ALT, alanine aminotransferase; APRI, aspartate aminotransferase to platelet ratio index; AST, aspartate aminotransferase; FIB‐4, fibrosis‐4 index; DM, diabetes mellitus; Hb, haemoglobin; HCC, hepatocellular carcinoma; HCV, hepatitis C virus; IFN, interferon; IQR, interquartile range; Plt, platelet count; SVR12, sustained virological response at 12 weeks; TSP2, thrombospondin 2; WBC, white blood cell count.

### 3.5 Cumulative HCC Occurrence Rate After DAA Therapy According to Other Clinical Scores at SVR12

**FIGURE 2:**
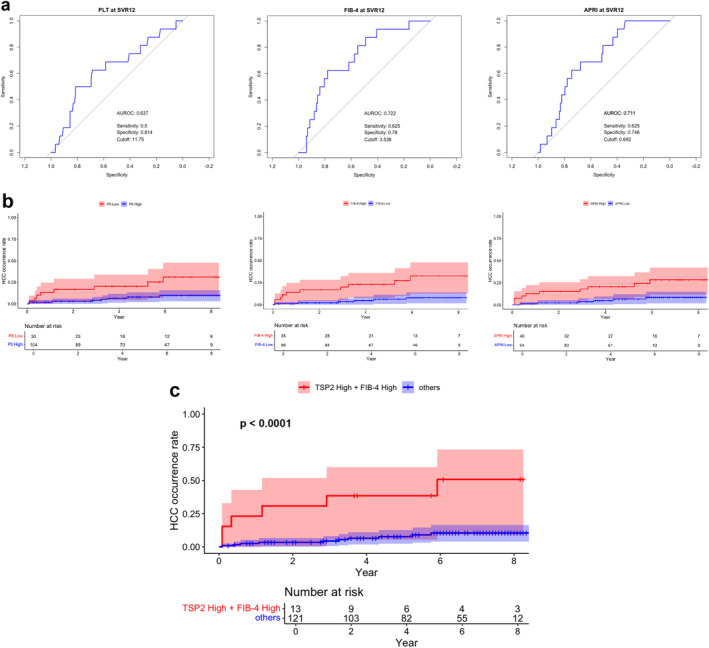
(a) Receiver operating characteristic analysis of prognostic markers for HCC occurrence after DAA therapy, including Plt, FIB‐4, and APRI at SVR12. (b) Cumulative HCC occurrence rate after DAA therapy, stratified by Plt, FIB‐4, and APRI at SVR12. (c) Cumulative HCC occurrence rate after DAA therapy, stratified by the combination of TSP2 and FIB‐4 at SVR12. TSP2 High: TSP2 > 32.12 ng/mL, Plt Low: Plt < 11.75 (×10^4^/μL), FIB‐4 High: FIB‐4 > 3.538, APRI High: APRI > 0.692. The ‘Year’ axis starts from SVR12. APRI, aspartate aminotransferase‐to‐platelet ratio index; AUROC, area under the receiver operating characteristic curve; DAA, direct‐acting antiviral; FIB‐4, fibrosis‐4 index; HCC, hepatocellular carcinoma; Plt, platelet count; SVR12, sustained virological response at 12 weeks; TSP2, thrombospondin 2.

The AUROC for predicting HCC occurrence after DAA therapy was 0.637 for Plt at SVR12, 0.722 for FIB‐4 at SVR12 and 0.711 for APRI at SVR12 (Figure 2a). The cut‐off value at SVR12 was 11.75 × 10^4^/μL for Plt, 3.538 for FIB‐4 and 0.692 for APRI, with sensitivity and specificity results at each cut‐off value being 0.5 and 0.814 for Plt, 0.625 and 0.78 for FIB‐4 and 0.625 and 0.746 for APRI, respectively.

Kaplan–Meier survival analysis utilising the respective cut‐off values for Plt, FIB‐4 and APRI at SVR12 revealed a significantly higher rate of HCC occurrence after DAA therapy in the Plt Low group (log‐rank *p* = 0.0050) as well as in the FIB‐4 High (log‐rank *p* = 0.0005) and APRI High (log‐rank *p* = 0.0033) groups (Figure 2b). These findings suggested that apart from TSP2, other clinical scores at SVR12 exhibited moderate predictive capability for HCC occurrence after DAA regimens.

### 3.6 Univariate and Multivariate Cox Proportional Hazards Models for HCC Occurrence After DAA Therapy

The results of the univariate Cox proportional hazards model for HCC occurrence risk after DAA therapy are summarised in Table 3. HCC history (hazard ratio [HR] 9.05, 95% confidence interval [CI] 3.36–24.38, *p* < 0.001), cirrhosis (HR 4.48, 95% CI 1.68–11.93, *p* = 0.003), Plt Low at SVR12 < 11.75 (HR 3.70, 95% CI 1.39–9.87, *p* = 0.009), FIB‐4 High at SVR12 ≥ 3.538 (HR 5.04, 95% CI 1.83–13.87, *p* = 0.002), APRI High at SVR12 ≥ 0.692 (HR 4.06, 95% CI 1.48–11.18, *p* = 0.007) and TSP2 High at SVR12 ≥ 32.12 (HR 3.96, 95% CI 1.47–10.64, *p* = 0.006) were all significantly associated with HCC occurrence after DAA treatment. Subsequently, to investigate factors associated with the development of HCC following DAA treatment, two multivariate Cox proportional hazard models were constructed, incorporating previous findings on HCC development and accounting for covariances between variables [19]. In Multivariate Model 1, the variables included age ≥ 65, male sex, history of HCC, FIB‐4 High at SVR12 and TSP2 High at SVR12. We found that male gender, history of HCC and TSP2 High at SVR12 were independent factors associated with HCC occurrence after DAA treatment (male: HR 4.84, 95% CI 1.62–14.43, *p* = 0.005; HCC history: HR 4.61, 95% CI 1.31–16.17, *p* = 0.017; TSP2: HR 3.93, 95% CI 1.39–11.06, *p* = 0.009). In Multivariate Model 2, where cirrhosis replaced FIB‐4 among the variables in model 1, male gender, history of HCC and High TSP2 at SVR12 remained independent factors for HCC development after DAA treatment (male: HR 4.09, 95% CI 1.40–11.89, *p* = 0.009; HCC history: HR 8.13, 95% CI 1.95–33.85, *p* = 0.004; TSP2: HR 3.64, 95% CI 1.24–10.63, *p* = 0.018). The concordance index (*C*‐index) for Multivariate Models 1 and 2 was 0.878 and 0.859, respectively, both indicating high predictive accuracy.

**TABLE 3:** Factors associated with HCC occurrence after DAA therapy.

	Univariate			Multivariate model 1			Multivariate model 2		
*C*‐index				0.878			0.859		
	HR	95% CI of HR	*p*	HR	95% CI of HR	*p*	HR	95% CI of HR	*p*
Age (≥ 65 years)	3.07	0.70–13.53	0.137	1.79	0.35–9.26	0.487	2.31	0.48–11.15	0.297
Male	2.42	0.88–6.66	0.088	4.84	1.62–14.43	**0.005**	4.09	1.40–11.89	**0.009**
DM	0.28	0.04–2.12	0.1						
Prior IFN	2.39	0.89–6.42	0.084						
HCC history	9.05	3.36–24.38	**< 0.001**	4.61	1.31–16.17	**0.017**	8.13	1.95–33.85	**0.004**
Cirrhosis	4.48	1.68–11.93	**0.003**				3.63	0.23–4.14	0.984
Plt Low	3.7	1.39–9.87	**0.009**						
(< 11.75 × 10^4^/μL)									
FIB‐4 High	5.04	1.83–13.87	**0.002**	2.59	0.68–9.86	0.161			
(≥ 3.538)									
APRI High	4.06	1.48–11.18	**0.007**						
(≥ 0.692)									
TSP2 High	3.96	1.47–10.64	**0.006**	3.93	1.39–11.06	**0.009**	3.64	1.24–10.63	**0.018**
(≥ 32.12 ng/mL)									

*Note:* Bold indicates a *p* value of less than 0.05. Abbreviations: APRI, aspartate aminotransferase to platelet ratio index; *C*‐index, concordance index; CI, confidence interval; DAA, direct‐acting antiviral; DM, diabetes mellitus; FIB‐4, fibrosis‐4 index; HCC, hepatocellular carcinoma; HR, hazard ratio; IFN, interferon; Plt, platelet count; SVR12, sustained virological response at 12 weeks; TSP2, thrombospondin 2.

### 3.7 Low Risk of HCC Occurrence After DAA Therapy in Patients Without TSP2 High + FIB‐4 High at SVR‐12

After DAA therapy for hepatitis C patients, it is crucial not only to pinpoint those at high risk of developing HCC, but also to identify low‐risk patients and establish suitable monitoring intervals. Accordingly, we investigated the feasibility of combining multiple markers to better recognise patients at low risk of developing HCC after DAA therapy.

As shown in Table 4, the TSP2 High + FIB‐4 High group at SVR12 displayed a sensitivity of 0.375, specificity of 0.941, positive predictive value (PPV) of 0.462, and negative predictive value of 0.917 for predicting HCC occurrence after DAA therapy. Compared with other prognostic markers, this group exhibited the highest specificity and PPV. Kaplan–Meier survival analysis revealed a significantly higher HCC occurrence rate after DAA treatment in the TSP2 High + FIB‐4 High group than in the others group (log‐rank *p* < 0.0001) (Figure 2c).

**TABLE 4:** Receiver operating characteristic analysis of prognostic markers at SVR12 for HCC occurrence.

	AUROC	Sensitivity	Specificity	PPV	NPV
TSP2 High + FIB‐4 High	—	0.375	0.941	0.462	0.917
Plt Low	0.637	0.500	0.814	0.267	0.923
FIB‐4 High	0.722	0.625	0.780	0.278	0.939
APRI High	0.711	0.625	0.746	0.250	0.936
TSP2 High	0.674	0.562	0.788	0.265	0.930

*Note:* TSP2 High: TSP2 ≥ 32.12 ng/mL, Plt Low: Plt < 11.75 (×10^4^/μL), FIB‐4 High: FIB‐4 ≥ 3.538, APRI High: APRI ≥ 0.692.

Abbreviations: APRI, aspartate aminotransferase‐to‐platelet ratio index; AUROC, area under the receiver operating characteristic curve; FIB‐4, fibrosis‐4 index; HCC, hepatocellular carcinoma; NPV, negative predictive value; PPV, positive predictive value; Plt, platelet count; SVR12, sustained virological response at 12 weeks; TSP2, thrombospondin 2.

## 4 Discussion

### 4.1 Main Findings

This study investigated the impact of DAA treatment on serum TSP2 levels and the role of TSP2 in carcinogenesis in patients with HCV. We observed a continuous decrease in serum TSP2 levels throughout DAA treatment, with TSP2 level at SVR12 emerging as an independently significant indicator for HCC development after DAA therapy. We also revealed that risk classification based TSP2 High + FIB‐4 High at SVR12 may identify a segment of patients with a low risk of HCC following DAA therapy.

### 4.2 Context With Published Literature

TSP2 is a glycoprotein secreted in the serum that is involved in cell–cell and cell–matrix interactions [6, 20]. Previous studies have demonstrated that serum TSP2 levels may serve as a biomarker for predicting advanced fibrosis and future HCC occurrence in nonalcoholic fatty liver disease patients [15, 16, 21, 22, 23]. Furthermore, we earlier showed that the THBS2 gene encoding TSP2 was highly expressed in HCV‐infected patients with advanced liver fibrosis and that serum TSP2 levels correlated significantly with histological scores of liver fibrosis and inflammation [7]. Therefore, the continued decrease in serum TSP2 levels with DAA treatment in this study is presumed to be a response to the quiescence of liver inflammation.

We recently revealed that THBS2/TSP2 regulated hepatic stellate cell (HSC) activation and collagen formation both in the presence and absence of transforming growth factor beta 1 [16]. This finding indicated that THBS2/TSP2 suppression in HSCs could potentially abrogate hepatic fibrosis, thus rendering it a promising target for fibrosis treatment [16]. On the other hand, THBS2/TSP2 expression has been implicated not only in liver fibrosis, but also in carcinogenesis. In pancreatic, gastric and breast cancers, TSP2 was suggested to play a role in tumour growth and be associated with prognosis [24, 25, 26, 27]. Another report has described the utility of TSP2 as a biomarker for HCC, which corroborates the present report [28]. Further basic and clinical investigations are imperative to elucidate the underlying mechanisms linking TSP2 to HCC.

Lastly, FIB‐4 is a useful marker for prognosticating the risk of HCC in patients who achieve a SVR after DAA treatment for HCV infection. Combining pre‐ and posttreatment FIB‐4 scores allows for a more detailed risk stratification of HCC development following a SVR [29]. Post‐SVR alterations in FIB‐4 scores correlate with changes in HCC risk, with decreases and increases associated with a lower and higher risk, respectively [29, 30]. Our multivariate analysis revealed TSP2 as an independent risk factor that was separate from FIB‐4. TSP2 may therefore be involved in HCC development through a mechanism different from that of FIB‐4. By combining TSP2 and FIB‐4, it appears feasible to identify individuals with a low risk of developing HCC. Specifically, patients without concurrently high TSP2 and FIB‐4 levels may have a particularly low risk of subsequent HCC.

### 4.3 Strengths and Limitations

The primary strength of this study is its demonstration that TSP2 at SVR12, a marker previously studied in liver disease and targeted for fibrosis treatment, may represent a predictive indicator for the future occurrence of HCC in DAA‐treated hepatitis C patients. Additionally, we revealed that patients who did not exhibit both TSP2 High and FIB‐4 High at SVR12 had a significantly lower risk of developing HCC after DAA therapy. However, our study has several limitations, including being retrospective in nature and limited in size. Since the subjects were uniformly Asian, future research of larger cohorts encompassing diverse ethnic backgrounds is warranted to validate our findings.

### 4.4 Future Implications

This study's findings on TSP2 in patients with HCV hold promising future implications. They suggest that monitoring serum TSP2 can aid in predicting HCC occurrence after DAA treatment. By combining TSP2 with FIB‐4, it may be possible to identify patients at a low risk of developing HCC post‐SVR, potentially allowing for a longer period between follow‐up visits compared with groups displaying other marker profiles. Further research is expected to enable the integration of TSP2 measurements into clinical practice towards earlier intervention and more personalised approaches to HCC surveillance and treatment in HCV‐infected individuals. In conclusion, TSP2 holds potential as a valuable biomarker for tailoring HCC monitoring in patients with hepatitis C who have undergone DAA.

## Author Contributions

T.K. and T.I. designed and performed the experiment and drafted the article. A.S., T.Y., T.O., S.‐i.W., H.K., Y.Y. and S.J. analysed patient clinical data. N.T. and T.U. supervised the research.

## Conflicts of Interest

The authors declare no conflicts of interest.

## Data Availability

The data sets generated and/or analysed during the current study are available from the corresponding author on reasonable request.
